# Data Gathering in Delay Tolerant Wireless Sensor Networks Using a Ferry

**DOI:** 10.3390/s151025809

**Published:** 2015-10-13

**Authors:** Mariam Alnuaimi, Khaled Shuaib, Klaithem Alnuaimi, Mohammed Abdel-Hafez

**Affiliations:** 1 College of Information Technology, United Arab Emirates University, P.O. Box 15551, Al Ain 00971, United Arab Emirates; E-Mails: k.shuaib@uaeu.ac.ae (K.S.); klaithem.nuaimi@uaeu.ac.ae (K.A.); 2 Electrical Engineering Department, College of Engineering, United Arab Emirates University, P.O. Box 15551, Al Ain 00971, United Arab Emirates; E-Mail: mhafez@uaeu.ac.ae

**Keywords:** ferry protocol, wireless sensor networks, delay tolerance networks, routing protocols, energy efficiency protocols

## Abstract

In delay tolerant WSNs mobile ferries can be used for collecting data from sensor nodes, especially in large-scale networks. Unlike data collection via multi-hop forwarding among the nodes, ferries travel across the sensing field and collect data from sensors. The advantage of using a ferry-based approach is that, it eliminates the need for multi-hop forwarding of data, and as a result energy consumption at the nodes is significantly reduced. However, this increases data delivery latency and as such might not be suitable for all applications. In this paper an efficient data collection algorithm using a ferry node is proposed while considering the overall ferry roundtrip travel time and the overall consumed energy in the network. To minimize the overall roundtrip travel time, we divided the sensing field area into virtual grids based on the assumed sensing range and assigned a checkpoint in each one. A Genetic Algorithm with weight metrics to solve the Travel Sales Man Problem (TSP) and decide on an optimum path for the ferry to collect data is then used. We utilized our previously published node ranking clustering algorithm (NRCA) in each virtual grid and in choosing the location for placing the ferry’s checkpoints. In NRCA the decision of selecting cluster heads is based on their residual energy and their distance from their associated checkpoint which acts as a temporary sink. We simulated the proposed algorithm in MATLAB and showed its performance in terms of the network lifetime, total energy consumption and the total travel time. Moreover, we showed through simulation that nonlinear trajectory achieves a better optimization in term of network lifetime, overall energy consumed and the roundtrip travel time of the ferry compared to linear predetermined trajectory. In additional to that, we compared the performance of your algorithm to other recent algorithms in terms of the network lifetime using same and different initial energy values.

## 1. Introduction

Recent improvements in hardware electronics technology have enabled manufacturers to develop low cost, low power and small size sensors [1,2,3]. Hundreds and thousands of these sensors are deployed as wireless sensor networks (WSNs) serving many applications based on the specific requirements of each one [4,5,6]. Nowadays, there are many applications of sensor networks covering different fields such as agriculture, medicine, military, environment monitoring, intrusion detection, motion tracking, machine malfunction, and many others [2]. Sensors can be deployed to continuously report environmental data for long periods of time.

In general, a wireless sensor network is a collection of static nodes with sensing, computation, and wireless communication capabilities [2]. Using mobile nodes as ferries to collect data from sensors in a WSN can improve the performance by increasing the lifetime of such a WSN and by maintaining coverage of the entire area. Ferries are mobile elements that are used to carry data and control information over distance to base stations or to a data center enabling more intelligent decisions to be made. They can also be used to connect isolated islands in WSNs. In addition, ferries can be used to resolve issues related to hidden nodes and the coverage of holes in a WSN which might have resulted from deployed fixed sensor nodes which have run out of energy.

In this paper, we propose a mobile ferry algorithm based on our previous published work, the Node Ranking Clustering Algorithm (NRCA) [7,8,9]. Using NRCA, the decision of selecting cluster heads in a WSN is based on their residual energy, their distance from the base station and an energy threshold that is used to replace cluster-heads. In this paper, the decision of selecting cluster heads is based on their residual energy and their distance from the ferry’s path composed of checkpoints (CPs). The checkpoint positions will be initially decided by deploying a virtual grid on the field and placing a checkpoint in the center of each grid. The checkpoints will then be changed based on the number of its attached cluster heads. The Travel Salesman Problem (TSP) will be used to find a Hamilton cycle to decide the path of the ferry. Checkpoints will represent the vertices and the distance between them will represent the edges. A cost function will be used to decide on which vertices will be visited first such that the overall cost will be minimized. Since TSP is NP hard [10,11] when the number of stops to make is larger than 4, a genetic algorithm will be used to choose the sequence of checkpoints to be visited. The main contribution of this algorithm is in finding an optimum (in terms of consumed overall energy and roundtrip travelling time) random path for the ferry to follow to collect data from the sensor network.

The rest of the paper is organized as follows: in Section 2, a survey on related work is summarized. Our proposed data collecting algorithm is described in Section 3. In Section 4, performance evaluation in terms of the network lifetime of the proposed algorithm is shown using different criteria. Finally, Section 5 concludes the paper.

## 2. Background Work

Using mobile ferries in WSNs is a relatively new area of research which is gaining the attention of many researchers. Incorporating ferries in WSNs helps in eliminating the need for multi-hop forwarding of data [12]. It also reduces the energy consumption at the nodes. However, using ferries might add delay in collecting, disseminating and processing data and thus might not be suitable for all applications. Existing research in this field can be classified in two main areas or categories as listed in the subsections below.

### 2.1. Path Determination

The paths that the ferry takes to collect sensed data from sensors can be classified into two categories: random paths and planned paths. Usually in the case of a random path the ferry is attached to people or animals moving randomly to collect sensed data whenever they are in the communication range of the static sensor nodes. In [13] mobile entities called mules were deployed in the environment. Mules pick up data from sensors when they are in close proximity, buffer it, and drop it off when within communication range of wired access points. The authors use a two-dimensional random walk to model the mobility of mules. Both mules and sensors are required to have memory capacity as they are buffering data. In [14] mobile nodes were used in the sensor field as forwarding agents. When a mobile node moves in close proximity to sensors, data is transferred to the mobile node for later depositing it at the destination. Analytical models were used to understand key performance metrics such as data transfer, latency to the destination, and power consumption.

Due to the random mobility of the ferry it is difficult to gather sensed data from all deployed nodes. Unlike the random path approach, in the planned path approach a path is determined before the ferry is dispatched and thus the ferry is sent to cover a certain area of deployed sensors to collect data. In [15], a wireless sensor network architecture for a traffic surveillance application with mobile sinks was proposed. All sensor nodes in this architecture were assumed to be located within direct communication range of the mobile sink. All multi-hop transmissions of high volume data over the network are converted into single-hop transmissions to further preserve energy. Therefore, nodes will transmit only in a single-hop fashion to the mobile sink.

In Mobi-Route [16], a routing protocol where the sink moves on a planned path to prolong the network lifetime in WSNs was proposed. In this protocol, the sink moves and stops at certain points of interest. The stopping time periods are designed to be long enough to allow for the collection of data. All deployed static sensor nodes need to be aware of the sink movement, its location and the length of stops to send any sensed data to it.

In [17] the authors proposed path-planning algorithms for an autonomous underwater vehicle (AUV) which acts as a mobile sink node for underwater sensor nodes. They used Value-of-Information (VoI) as the metric for choosing the path of the AUV. The VoI serves as a marker for evaluating the quality of information with respect to the time spent collecting that data.

The authors in [18] used a single ferry to collect data from a circular dense sensor network. They showed that the optimal mobility strategy of the ferry was achieved when moving at the border of the sensing area. They divided the area into circles starting from the source. The inner circles forward the data to the outer ones until the border was reached, where the ferry was used to collect the sensed data. Thus multi-hop forwarding was used to finally reach the ferry. In [19,20] the ferry visits exact rendezvous points to collect data. These points buffer and aggregate data to the ferry from the nodes though multi-hop forwarding. In [21] the so-called weighted rendezvous points (WRP) algorithm was proposed, where nodes are used as rendezvous points. Cluster heads and nodes send their collected data to these points though multi-hop forwarding. The tour path of the ferry to these points is built by assigning a weight to each one represented by the distance in number of hops from the path and the number of data packets each node is forwarding to the closest point. In [22] the authors chose cluster heads with the highest energy as rendezvous points and then built the tour of the mobile sink to these energy rich cluster heads to collect data. In Section 4.6 we compare our FNRCA against the WRP algorithm and the one in [22].

Our proposed approach is different from the above as the ferry does not have to visit each node in the network to collect sensed information. Instead, the sensing field area will be divided into virtual grids and a checkpoint will be placed in each grid. The ferry will only visit these checkpoints to collect data. Our approach also uses TSP and genetic algorithm to choose the optimum path of the ferry which consists of visiting a list of sequenced checkpoints. The sequence of checkpoints that will be visited will be decided by assigning a weight to each checkpoint and deciding which checkpoint will be visited first. Moreover, the NRCA algorithm will be applied in each virtual grid to decide on the best placement position for each checkpoint in order to preserve the energy of the whole network. Our aim is to minimize the overall round trip travelling time of the ferry and to minimize consumed energy in the network. This is achieved by modifying NRCA to be applied in reference to the position of the checkpoint rather than the position of the base station *i.e*., the sink. By doing this, each checkpoint will act like a virtual sink within each virtual grid. We refer to the new modified NRAC algorithm as ferry based NRCA or FNRCA. In FNRCA distance used to rank the nodes is in reference to the checkpoint position rather than the position of the base station as was done in NRCA.

### 2.2. Scheduling the Dispatch of the Ferry

The scheduling of when exactly to send the ferry to collect sensed data from nodes is a complicated task. In [23,24] the authors studied the scheduling problem where the path of the mobile sink was optimized to visit each node in the WSN before its buffer gets full. Buffer overflow was used as a trigger to send the ferry to collect data to avoid data loss.

In [19,20] the authors suggested the mobile sink to visit exact locations (rendezvous points) based on a predetermined schedule to collect data. The rendezvous points buffer and aggregate data originated from the source nodes through multi-hoping and transfer it to the mobile sink upon its arrival.

The authors in [25] used a ferry to help in collecting data in partitioned wireless sensor networks and transfer the collected data stored locally back to the base station. The authors classified scheduling of a ferry visit into three categories: time-based scheduling, location-based scheduling and dynamic-based scheduling. Time-based scheduling happened when a node dies and its death leads to partitioned WSNs. This node will have a higher priority for ferry visits. Location-based scheduling assigns the nodes closer to the base station a higher priority for the ferry visit. Dynamic-based scheduling is based on calculating the distances between the current location of the ferry and the locations of the partitioned wireless sensor networks that have not been visited by the ferry, and selects the shortest distance for the next visit.

In [26] the authors considered on-demand data collection where sensor nodes broadcast data collection requests when their buffers are about to be full. On receiving such requests, the ferry moves toward the sensor nodes to collect data and transfer the data to the sink.

The authors in [27] proposed a mobile node to help disseminate data to the sink. It was used to move back and forth along the linear network, and collect data from the individual sensors when they come within its communication range. The mobile node will then transfer the collected data to a base station. The mobile node was also used to perform other functions, such as data processing, and aggregation, and can also transport messages from the sink to the sensor nodes.

## 3. Ferry Node Ranking Clustering Algorithm (FNRCA)

Since data transmission can account for up to 70% of the power consumed by typical sensor nodes [28,29,30,31], substantial energy can be saved by reducing the distance messages are transported over and the amount of data transmitted to the base station. The distance of the nodes from the base station and inter-node distances can have a high impact on nodes’ energy consumption and thus in prolonging the network lifetime. Network lifetime can be defined either as the time for the first node to die, the time for the last node to die or the time for a certain percentage of nodes in the WSN to die [32]. Moreover, in dense deployments of sensor nodes in a WSN, nodes can cooperate to send data and therefore distribute the energy consumption between them.

In this paper a proposed ferry-based node ranking clustering algorithm (FNRCA) is used to collect data from nodes. The difference between this algorithm and other algorithms is that this one uses a more efficient mechanism to select cluster heads (CHs). This is done by considering the nodes’ distances and locations within the sensing field, current energy levels of nodes and by calculating the number of rounds that each node can be a cluster head for, to maximize the network lifetime and decrease excessive communication overheads used for electing new cluster heads. In this algorithm, nodes are ranked based on their current energy level (En) and their positions (Dn) with reference to the predetermined checkpoints on the mobile ferry’s trajectory. This ranking is used for choosing cluster heads which are also ranked into levels based on their position, the Euclidean distance from the ferry’s checkpoints. Therefore, each node is assigned a rank Rn (En, Dn) reflecting its candidacy for it being elected as a cluster head.

**Figure 1:**
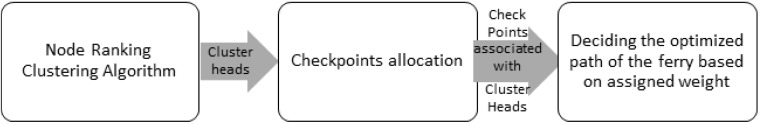
Illustration of phases used by FNRCA.

In our algorithm, once the ferry reaches a checkpoint, it broadcasts a notification message to all nodes in its communication range informing them of its presence at the respective checkpoint they are associated with. Nodes within each cluster will then start sending to their associate cluster heads any sensed data to be carried to the base station. The ferry stopping time at each checkpoint is determined by the number of attached cluster heads to that particular checkpoint as will be shown later. Using this strategy, cluster-heads will not have to worry about the speed or the direction of the ferry and energy wasted in doing this will be preserved. The algorithm also is provided with efficient energy management strategy where cluster heads are only waking up when the ferry sends them a notification massage to inform them of its presence. The idea of using the ferry to collect data further preserved energy by reducing multi hopping which consumes energy. To optimize the ferry’s path, a weight was assigned to each checkpoint to be able to choose the best sequence, the order of checkpoints to be visited and the needed stopping time at each one. This eliminates the loss of messages due to any wrong prediction of the position of the ferry or its movement. Our algorithm uses three phases as shown below in Figure 1.

The proposed algorithm is shown to be energy efficient because it aims to minimize the energy consumed in the network to collect and transfer data to the BS using a mobile ferry. In the next subsection we introduce the proposed algorithm in more details.

### 3.1. Node Ranking Clustering Algorithm

In most previously proposed clustering algorithms a node is elected as a cluster head either randomly or based on having the highest residual energy in a cluster. This selection might lead to inefficiencies [33]. One of the common drawbacks of these algorithms is taking a longer path, number of hops, to the base station. The additional number of hops data needs to travel to get to the base station will cause more energy consumption. Also, there can be forgotten nodes or disconnected nodes which are not covered by any of the cluster heads chosen due to being far from any reachable one. Moreover, frequent replacement of cluster heads in each sensing round wastes energy. These problems can be avoided in our proposed algorithm where data can be sent through the correct path or direction with respect to a mobile ferry’s path consisting of checkpoints and by the BS maintaining a global knowledge of all nodes in the WSN area to ensure that all alive nodes are connected through the proper choice of cluster heads. Using a ferry to collect sensed data and control traffic data from cluster heads results in a decreased traffic forwarding burden on cluster heads in the network as the number of multi hop forwarding attempts is reduced. In addition, we propose the use of an energy threshold technique in making decisions to replace cluster heads which prolongs the lifetime of nodes especially those closer to the ferry’s checkpoints. This in effect prolongs the overall network lifetime as nodes closer to the ferry’s checkpoints are more critical than those far away nodes in maintaining connectivity in the network.

In the proposed algorithm several assumptions are needed: first, the Base Station (BS) is placed at a fixed position and has unlimited energy. Thus no constraints are assumed with regards to power consumption due to data processing and communication. Second, all nodes are assumed to have the same energy level at the set up phase, which is known to the BS. Third, the sensing field dimensions are also assumed to be provided to the BS. Fourth, it is assumed that the mobile ferry is dispatched from the base station and returns back to it once its task is completed. In addition, it is assumed that there are no energy constraints with respect to the ferry which is assumed to be moving at a fixed speed. Nodes throughout the sensing field are randomly and uniformly distributed.

### 3.2. Description of the Algorithm

The proposed algorithm is an extension based on our previously published work NRCA [7,8,9] with node ranking being based on the planned path of the ferry rather than the location of the BS. The following steps give a description of the algorithm and cluster heads’ selection process:

- The BS divides the sensing field into smaller partitions called clusters based on the assumed communication/sensing range of the nodes.
- The sensing field will then be divided into virtual square grids based on the specified sensing range. Each virtual grid will be of size r× r where r is the sensing range. Multiple clusters fall within one or more virtual grids.
- Initially, a ferry checkpoint (virtual base station) is placed at the center of each virtual grid.
- Initially NRCA is used to choose CHs based on their location from the ferry’s checkpoints.
- Nodes and cluster heads will associate themselves with the ferry’s checkpoint based on their location within each virtual grid.
- Border line nodes and cluster heads will be associated with cluster heads and checkpoints closer to them based on distance respectively.
- After the initial phase, NRCA is applied on each virtual grid based on the position of the ferry’s checkpoint and energy values of the associated nodes. Therefore, the energy consumed per virtual grid will be minimized. This is explained below in the subsequent sections.
- The ferry will be dispatched from the BS to visit all checkpoints and return back to the BS using a Hamilton cycle as will be shown later.
- At each checkpoint the ferry stops to collect gathered data from cluster heads associated with this checkpoint. Gathered data consists of sensed data and control information like nodes energy values and nodes GPS locations.
- Dissemination of data from cluster heads to the ferry is triggered by a control message communicated by the ferry to the cluster heads associated with each checkpoint. The time the ferry will stay for at each checkpoint is determined based on several parameters as will be shown later.
- In the subsequent rounds of dispatching the ferry, the BS choses the new locations of the checkpoints based on the collected information to minimize the energy of the overall sensing field as will be shown later. The BS will then determine the new path of the ferry using Hamilton cycle as was done in the initial phase.
- The energy model used for sensing and dissemination of data in our simulation is the same used by [33,34,35,36,37,38].

### 3.3. Cluster Head Selection Process

After the initial formation of clusters and based on the information collected through the first dispatching round of the ferry, nodes in each cluster are ranked by the BS based on their position with respect to the checkpoint they are attached to and on their current energy level. This information is dispatched back to the nodes through the next ferry visit. Nodes with the maximum residual energy and minimum distance will be chosen as a cluster head based on NodeRanking (En, Dn)  where:

(1) $$[Dn\left(i\right)=Min\left(D\left(i,Closest_{CP}\;\right)\right)\;,\left(En\left(i\right)\right)=Max\left(ResidualEnergy\left(i\right))\right]$$

where:

(2) $$│D\left(i,Closest_{CP}\right)│=\sqrt{{\left(Xi-Xcp\right)}^{2}+{\left(Yi-Ycp\right)}^{2}}\;$$

and ResidualEnergy (En (i)) is the current energy level of node i, $D\left(i,\;Closest_{CP}\right)$ is the Euclidean distance of node i to the closest checkpoint. Given a particular deployment region of interest, $X_{i}\;$ and Yi are the X and Y positions of node i. Xcp and Ycp are the X and Y positions of the closest checkpoint on the sensing field.

A cluster head in each cluster will be changed when its energy level reaches a pre-defined threshold or a calculated value and not every sensing round. This will make it possible for ith node to continuously play the role of a cluster head for multiple sensing rounds and thus save any wasted energy for control and exchanged messages used in replacing it. In the next two subsections we will discuss more the placement of the checkpoints and the stopping time the ferry will spend at each checkpoint.

### 3.4. Ferry Checkpoints Locations

To decide the location of the ferry’s checkpoints, we first create a virtual grid based on the specified sensing range. Each virtual grid will be of size r × r. A checkpoint will be initially placed in the center of each square in the virtual grid. Then NCRA will be applied on each square in the grid where nodes will be ranked according to their energy levels and their distance from the checkpoints. After the first round of the ferry, the checkpoints positions will be changed in each grid by the BS based on the collected related information by the ferry in the first dispatched round. Each checkpoint in each virtual grid will be placed closer to the larger number of neighbouring cluster heads. The checkpoint coordinates, Xcp(j) and Ycp(j), in each virtual grid are calculated by the following equations:

(3) $$\text{Xcp}\left(\text{j}\right)=\frac{1}{{\text{N}}_{\text{j}}}\overset{N_{\text{j}}}{\underset{\text{k}=1}{\sum }}{\text{X}}_{\text{k}}^{\text{j}}\;$$

(4) $$\text{Ycp}\left(\text{j}\right)=\frac{1}{{\text{N}}_{\text{j}}}\overset{N_{\text{j}}}{\underset{\text{k}=1}{\sum }}{\text{Y}}_{\text{k}}^{\text{j}}\;$$

where ${\text{N}}_{\text{j}}$ is the total number of attached cluster heads associated with the same checkpoint.

Following is the pseudo code for choosing the checkpoint location in each grid:

○ **Input**: a subset of cluster heads CPch in each virtual grid, the virtual grid dimensions and the sensing range r between the ferry and clusters heads;
○ **Output**: if the subset of all cluster heads which can be covered by a circle with a radius at most r, return the circle’s center (Equations (3) and (4)) or false otherwise and no change in the checkpoint position *i.e*., it will be its previous position.
○ **if**
○ radius > r **then**
○ **return** false;      // no change in checkpoint position
○ **else**
○ center(x, y) = (Equations (3) and (4))
○ **return** center. // checkpoint position will be the center (x, y)
○ **end if**

### 3.5. Stopping Time of the Ferry at Each Checkpoint

The stopping time (ST) is the time period the ferry will stay at each checkpoint, j, to allow the associated cluster heads to send gathered data to the ferry. This time period depends on the number of associated cluster heads, their buffer sizes and the transmission time of a bit based on the assumed medium physical characteristics:

(5) ST (j)=BuffSize(CHj) × NumberOfAttachedCHs ×TimeToTransmitAbit+T

where BufSize is the cluster head memory size in bits, the NumberOfAttachedCHs is the number of cluster heads associated with checkpoint j, TimeToTransmitAbit is the time needed to transmit a bit of information to the checkpoint and T is an assumed constant delay added to account for propagation delay.

### 3.6. Problem Formulation

Given a set of cluster-heads **CHs**$\left[CH_{i},i=1,..,n\right]$ and a set of checkpoints **CPs**$\left[CP_{j},\;j=1,..,m\right]$ in a multi hopping WSN, the dispatched ferry need to move along a path to collect data from associated nodes when stopping at the checkpoints return back to the base station while satisfying the following two main goals:

*Goal one*: Minimizing the overall tour time of the ferry (delay):

(6a) $$\text{Tour}\_\text{Time}\left(\text{s}\right)=\text{Travel}\_\text{Time }\left(\text{s}\right)+\sum_{\text{j}=1}^{m}{\text{ST}}_{\text{s}}\left(\text{j}\right)\;$$

(6b) $$Tmax=\text{Max}(\text{Travel}\_\text{Time }\left(\text{s}\right)+\overset{m}{\underset{\text{j}=1}{\sum }}{\text{ST}}_{\text{s}}\left(\text{j}\right)\;)\;$$

such that:

(7)  Tour_Time(s) <Tmax 

where s = 1,2,3… corresponds to the round of data collection, Tour_Time is the total round trip travelling time of the ferry from the base station to each checkpoint j plus the stopping time at each check point of rounds:

(8) $$\text{Travel}\_\text{Time }\left(\text{s}\right)=\frac{{\text{D}}_{\text{s}}\left(\text{BS},\text{ CP}1\right)+\sum_{\text{j}=1}^{\text{m}-1}{\text{D}}_{\text{s}}\left(\text{CP}\left(\text{j}\right),\text{ CP}\left(\text{j}+1\right)\right)+{\text{D}}_{\text{s}}\left(\text{CP}\left(\text{m}\right),\text{BS}\right)}{\text{Ferry}\_\text{Speed}}$$

where m is the total number of checkpoints, n is the number of cluster heads, Travel_Time is the round trip travelling time of the ferry from the base station to each checkpoint j and returning back to the base station. $D_{s}\left(BS,\;CP1\right)$ is the distance from the base station to the first checkpoint and $D_{s}\left(CP_{m},BS\right)$ is the distance from the last checkpoint visited by the ferry to the base station. Ferry_Speed is the assumed fixed speed of the ferry.

*Goal two*: Minimize the overall energy consumption of the network by applying our FNRCA in each virtual grid *i.e*., finding the:

(9) $$Min\overset{\text{n}}{\underset{\text{i}=1}{\sum }}\text{E}\left(\text{i}\right)\;$$

and by minimizing the sum distance from the checkpoints and their associated cluster heads as:

(10) $$Min\overset{m}{\underset{cp=1}{\sum }}\overset{\text{n}}{\underset{\text{ch}=1}{\sum }}\text{D}\left(\text{ch},\text{ cp}\right)$$

such that:

(11) ∀ ch ∈ CHS, ∃ cp ∈ CPS : D(ch, cp) ≤ r

where n is total number of cluster heads, ch is a cluster head  CHS is the cluster heads list, cp is a checkpoint and CPS is the list of checkpoints.

In order to choose the optimum path of the ferry and achieve the above goal and constraints a weighting scheme is used to order the checkpoints into the sequence to be visited by the ferry.

### 3.7. Checkpoints Weighting Scheme

To determine the path of the ferry or which checkpoints to visit first, a weighting scheme is used based on determining the following weights:

- Checkpoints with larger number of attached cluster heads:

Checkpoints with larger number of attached cluster heads will contribute more to the amount of the data collected and in order to reduce data lost, they will have a higher priority of being visited first by the ferry. The weight for such a CP, j, is calculated as:

(12) $${\text{W}}_{1}\left(\text{j}\right)=\frac{\text{Attached}clusterheads\left(j\right)}{N_{T}}\;$$

where $N_{T}$ is the total number of cluster heads in the network.

- Checkpoints closer to the Base Station:

Checkpoints closer to the Base Station will be given a higher weight in order for the ferry to start the collection process from them first and then move ones further away. Their weight will be calculated as:

(13) $${\text{W}}_{2}\left(\text{j}\right)=\frac{1}{D\left(\text{CP}\left(j\right),\;BS\right)}\;$$

- Checkpoints closer to each other:

Checkpoint closer to each other will reduce the traveling time and distance of the ferry; therefore, they will have a higher priority of being visited first by the ferry and their weight will be calculated as:

(14) $${\text{W}}_{3}\left(\text{j}\right)=\frac{1}{\min\left[D\left(CP\left(j\right),CP\left(l\right)\right)\right]},\;l=1,2,..m,l\neq j\;$$

The overall weight (W) is computed as:

(15) $$\max W=\overset{m\;}{\underset{j=1}{\sum }}W_{1}\left(j\right)+W_{2}\left(j\right)+W_{3}\left(j\right)$$

Following is the pseudo code for ordering the checkpoint in Travelling Salesman Problem sequence to be visited by the ferry according to the weight given to them:

○ **Input**: a set of checkpoints, their attached cluster heads.
○ **Output**: A sequence of checkpoint for the ferry to follow.
○ //Optimal Travelling_Salesman_Problem_tour
○ **while** there exist checkpoints **do**
○ **for all** CPj (j = 1, 2, ..., m − 1) **do**
  find the weight W from Equation (15).
○ **End for**
○ Select CP with maximum weight
○ Add it to the TSPtour list {CPj,CPj + 1…}
○ Remove it from the set
○ **end while**
○ **return** TSPtour

The output of the above pseudo code which is the ordered sequence of checkpoint will be used as an input to the next pseudo code of the genetic algorithm. In our work, TSP will be used to find a Hamilton cycle to decide the path of the ferry, where checkpoints represent vertices and the distance between them will represent the edges. The above weight will be used to choose which vertices will be visited first such that the overall consumed energy and roundtrip travelling time will be a minimum.

Assuming a directed graph (G) with weights on the edges where G = (Vertex, Edge) we will find a Hamilton cycle where the cycle covers all the vertices only once and seeks a minimal weight subset of edges.

The abovementioned problem can be solved easily in a short time if the number of checkpoints are four or less by trying all possible paths (4!) and finding the minimum weight among them. However, if the number of checkpoints is more than four there will be permutations of possible paths which will take much longer time and processing capabilities. Therefore, we used a genetic algorithm to find the best path based on our own fitness function, goals and weights as shown in the below subsections.

### 3.8. Applying a Genetic Algorithm to Elect a Path

Since TSP is an NP-hard problem [10,11,38] we used a genetic algorithm to find the optimum sequence of checkpoints to be visited by the ferry. Genetic Algorithms are heuristic approaches which can be used to solve the TSP. They use simple chromosomes to encode solutions of data and apply crossover and mutation operators to these chromosomes to find an optimum solution. Good solutions will be selected by the fitness function and reproduced to produce a better solution and the bad ones will be removed. After several generations the genetic algorithms will produce an optimum solution to the problem.

In this work, we represented the ferry’s path as a list of genes or chromosomes. Where each checkpoint will have a number to identify it. e.g., (1, 2, 3, 4….. CPm) and the path or the solutions will be represented by the ordered sequence of checkpoints. Zero is used to represent the Base Station. The path will start and stop at the Base Station, so each path will contain 0 at the start and end of its sequence. Example of path representation will be (0,3,1,2,4,0). Below is the Pseudo code for the used genetic algorithm, the input to this pseudo code will be taken from the previous pseudocode that ordered the checkpoints into sequence according to their weights to be visit by the ferry.

○ **Input:** p(t) and c(t) are parents paths and offspring candidate paths in current generation t.
○ // Input will be taken from the previous pseudocode
○ **Output:** The optimum solution TSP.
○ T←0;
○ Initialize p(t);
○ Evaluate p(t);
○ **While** (there exist p(t)) **do**
○ Perform crossover and mutation p(t) to get c(t);
○ Evaluate c(t) with the fitness function(c(t));
○ Select p(t + 1) from p(t) and c(t);
○ T ← t + 1;
○ **End While**
○ **End**

#### 3.8.1. Crossover Operation

We used ordered crossover (OX) in our genetic implementation which was used in BERLIN52, the best known program for TSP so far [11,38]. Given two parent chromosomes, two random crossover points are selected partitioning them into left, middle and right portions. Then child inherits its middle section from parent1, and its left and right portions is determined by their order and position from the second parent. An example of ordered crossover is shown below:

Given the following two paths:

1st Path = (1 2 3 4 5 6 7 8 9)

2nd Path = (4 5 2 1 8 7 6 9 3) 

Based on the used OX genetic implementation [11,38], the following steps are performed:

1. Partition each path into three segments (left, middle, right)
  1st Path = (1 2 3 | 4 5 6 7 | 8 9)
  2nd Path = (4 5 2 | 1 8 7 6 | 9 3)
2. Copy the middle segment of both paths, the two candidate paths become as follow:
  1st candidate path = (- - - | 4 5 6 7 | - -)
  2nd candidate path = (- - - | 1 8 7 6 | - -)
3. Reorder each of the sequences starting from the right segments according to their order in the second path without repeating the already copied numbers (9-3-2-1-8). As 4, 5 7 and 6 are already copied.
4. Place the ordered sequence into the path starting from the right segment.
  1st candidate path = (2 1 8 | 4 5 6 7 | 9 3)
5. Generate new candidate paths as:
  1st candidate path = (2 1 8 | 4 5 6 7 | 9 3)
  2nd candidate path = (3 4 5 | 1 8 7 6 | 9 2)

#### 3.8.2. Mutation Operator

The resulting children from ordered crossover operation will now be subjected to the mutation operator in the final step to form a new generation. This operator randomly flips or alters one or more bit values at randomly selected locations in a chromosome. An example is shown below where 8 has been altered to 9:

Path 1       =   (1 2 3 4 5 6 7 [8])
Candidate path 1   =   (1 2 3 4 5 6 7 [9])

For implementing mutation in MATLAB we used the “*MutationFcn*” command.

#### 3.8.3. Fitness Function

The fitness function is used to measure the goodness of the produced children in terms of predefined goals, where bad solutions will be eliminated and good solutions will be kept. Our two goals, as shown in Equations (7) and (9), are: first, to evaluate the total travelling time of the ferry and second, to evaluate the total energy consumed in the whole network subject to the constraint in Equation (11). Based on the first goal, the fitness function “*Time_Fitness_Fun*” in Equation (16) will evaluate the travelling time where the shorter the travelling time the better the path will be, however if the travelling time is greater than Tmax, it will be eliminated by assigning a negative value to the function represented by, −∞, to exclude this solution from the solutions set. From the second goal, the smaller the total energy consumed the path gives, the fitter the solution will be. Such paths will be preserved to be used to reproduce a better solution.

For the implementation of the fitness functions we used the MATLAB “fitnessfcn” command given by:

(16) $$Time\_fitness\_Fun=\{\begin{array}{c} Tour\_Time\left(s\right),\;Tour\_Time\left(s\right)<Tmax\\ -\infty \;,\;Tour\_Time\left(s\right)\geq Tmax \end{array}$$

(17) $$Energy\_fitness\_Fun=\;\overset{\text{n}}{\underset{\text{i}=1}{\sum }}\text{E}\left(\text{i}\right)\;$$

## 4. Performance Evaluation

**Table 1:** Parameters used in the simulation, values for the various energy parameters are per the energy model used by [33,34,35,36,37,38].

Notation	Description
N = 400	Total number of sensor nodes
Eo = 0.5J/node	Initial energy of each node
Eelec = 50nJ/bit	Per bit energy consumption
EDA = 5nJ/bit	Energy for data aggregation
Eamp = 100 pJ/bit/${\text{m}}^{2}$	Amplifier transmitting energy
Area = 200 × 200	Area used in the simulation in meters
# Checkpoints	Varies according to the sensing range and the area : Area/sensing Raduis r
Packet size	256 bits
Data Rate	256 Kbps
Sensing Radius: r	50 m, Zigbee has a max of 100 m
Buffer size	256 K Bytes
Tmax	Time of the longest tour of the ferry`
Ferry_speed	100 m/min

To evaluate the performance of the proposed FNRCA algorithm we used MATLAB to simulate the algorithm on a 200 m × 200 m sensing field. Table 1 shows the parameters used in this simulation environment which are standard parameters used by all researcher in this field. Every node was given an initial energy of 5 J. The energy for data aggregation is 5 nJ/bit. The energy to run the radio is 50 nJ/bit. The amplifier transmitting energy is 100 nJ/bit/${\text{m}}^{2}$. The packet size is 256 bits. The data rate is 256 kbps. Ferry speed is 100 m/ min which represent fast walking. Using simulation, we considered the network lifetime, energy consumed and the total time of one tour of the ferry metrics to evaluate the performance.

### 4.1. Simulated Scenarios

**Figure 2:**
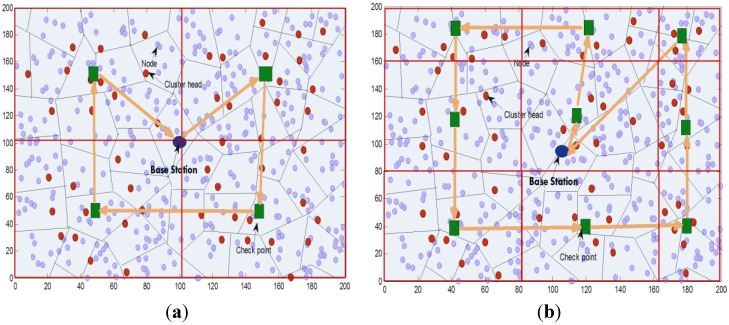
(**a**) Path of the ferry where four checkpoints are used; (**b**) Paths of the ferry with checkpoints, where nine checkpoints are used.

As shown in Figure 2a and b, the ferry will move along a nonlinear path dispatching from the Base Station which will be across the center of the sensing field. It visits each checkpoint only once per round to collect the data from cluster-heads and carry this data back to the BS. We showed in our previously published work [39] that centered predetermined path outperformed the diagonal path in terms of network lifetime and energy consumed. In the shown figure, four checkpoints (Figure 2a) and nine checkpoints (Figure 2b) are used.

To evaluate the performance we looked at network lifetime, energy consumed and the time of the overall roundtrip as will be shown in the subsections below. We ran the simulation five times and took the average of the runs to present our results.

### 4.2. Performance Based on Network Lifetime

Network lifetime is defined here as the time interval from the time the sensor network starts its operation until the death of the last node in the network. We compare the performance of using a four checkpoints TSP with a genetic algorithm, referred to as the optimized path, to the case of using a predetermined fixed path in the center of the sensing field and the multi-hop NRCA without the use of a ferry. From Table 2, we can see that the last node died in NRCA at round 3311 making it the least achiever with the shortest network lifetime among the other two. On the other hand, we can see that the optimized nonlinear path based on TSP with a genetic algorithm had the longest network life time as its last nodes died at round 4003 compared to the predetermined path where the first node died at round 1763 and the last at round 3830.

**Table 2:** Simulation results for the network lifetime based on Figure 1a.

Protocols	Measurements	
	*Round First Node Dies*	*Round Last Node Dies*
Optimized path	2010	4003
Predetermined path	1763	3830
NRCA	1300	3311

### 4.3. Performance Based on Energy Consumed

**Figure 3:**
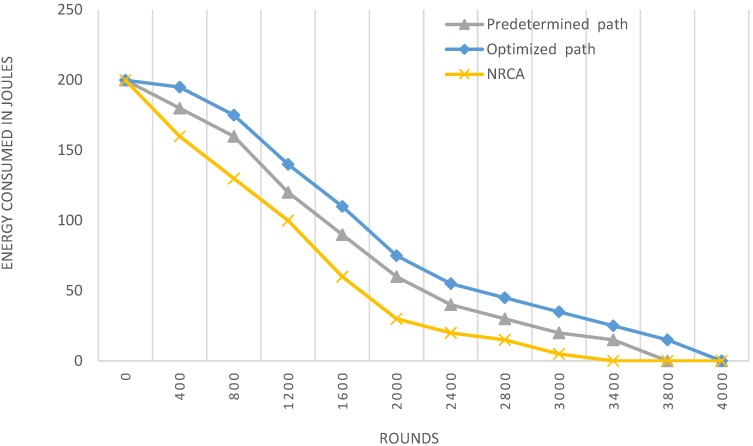
Energy consumption in the network.

As shown in Figure 3 the energy consumed per round in the optimized path case are less than the predetermined one and NRCA. Dividing the region into virtual grids with checkpoints in each one helps in reducing the energy consumption in these grids and as a result prolongs the lifetime of cluster heads and preserves the overall energy of the whole network.

### 4.4. The Overall Time of One Round Trip of the Ferry

The total overall time of one round trip of the ferry is defined as the overall travelling time of the ferry from the base station to each checkpoint and returning back to the BS plus the stopping time at each checkpoint, once, to collect data. As shown in Table 3, the predetermined path with four checkpoints took around 5.40 min per one round of data collection while 4 min were recorded for the optimized path.

**Table 3:** Simulation results for one round collection.

	Predetermined Path	Optimized Path
Time in minutes	5.40	4

### 4.5. Changing the Number of Checkpoints

By changing the sensing range for the optimized path, the number of checkpoints will be changed as well. We changed the sensing range to be 20 m and the number of checkpoints to be 25 checkpoints, 40 m and the number of checkpoints to be nine checkpoints, 50 m and the number of checkpoints to be four and finally, to be 100 m and the number of checkpoints to be one. Table 3 shows the network performance of changing the sensing range and the number of checkpoints. From Table 4 we can see that the network lifetime increases as the number of the checkpoints increases. This is because the more checkpoints we have, the less distance data will travel which in returns saves cluster heads energy and the overall energy of the network. However, looking into the overall time of one round of data collection of the ferry, we can see from Table 5 and Figure 4 that it is increases as the number of checkpoints increases. Roundtrip travelling time has a direct relationship with the number of checkpoints. This is due to the increase in the length of the traveling path plus the increase in the number of stopping time at each checkpoint. Given a particular application, the number of checkpoints can be chosen for a particular scenario based on the maximum tolerable delay.

**Table 4:** Simulation results for the network life time using different number of checkpoints.

#Checkpoints	Measurements	
	Round First Node Dies	Round Last Node Dies
Sensing range 20 #Checkpoint 25	2460	4433
Sensing range 40 #Checkpoint 9	2111	4120
Sensing range 50 #Checkpoint 4	2010	4003
Sensing range 100 #Checkpoint 1	1400	3500

**Table 5:** Simulation results for one round collection.

#Checkpoints	Time in minutes
Sensing range 20 #Checkpoint 25	18.60
Sensing range 40 #Checkpoint 9	9.60
Sensing range 50 #Checkpoint 4	5.40
Sensing range 100 #Checkpoint 1 which is the base station	3

**Figure 4:**
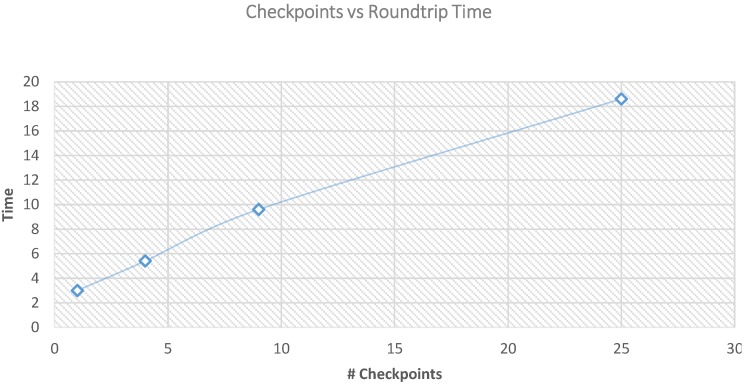
Number of checkpoints *vs.* round trip time.

### 4.6. Performance Evaluation of FNRCA against other Algorithms

**Figure 5:**
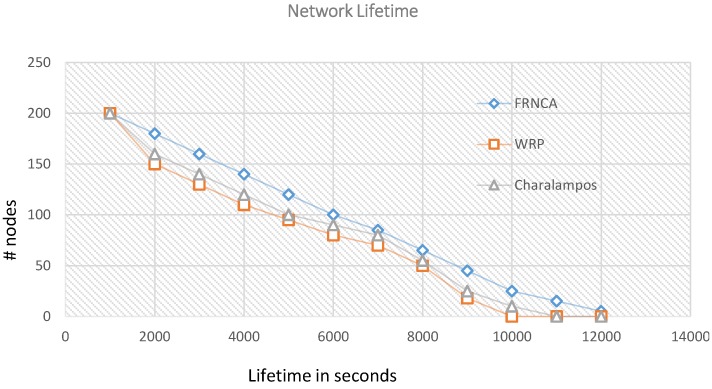
Network Lifetime for FNRCA, WRP and Charalampos using different initial energy values.

**Figure 6:**
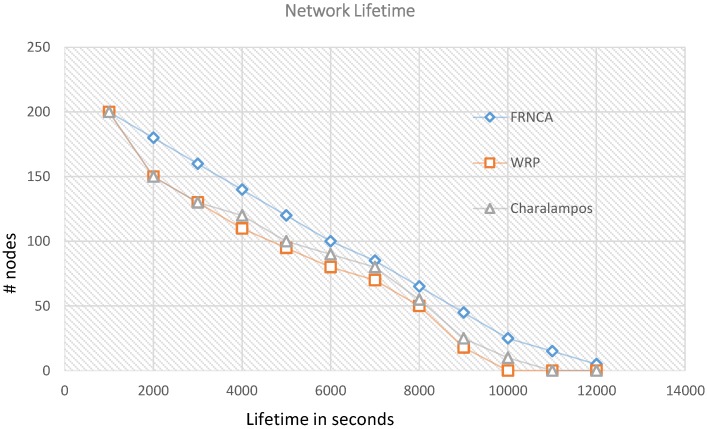
Network Lifetime for FNRCA, WRP and Charalampos using the same initial energy values.

We looked into comparing our algorithm with other state of the art ones under not exact but similar operating conditions. We are using a random uncontrolled path, but in the literature, some of the well-known algorithms we found such as [40]) are using controlled paths for the ferry to follow. In addition, there are other algorithms which are using multiple mobile sinks such as in [19,20,40] while we are using only one mobile object which is the ferry as a temporary sink. Moreover, we are limiting the multi-hoping in our algorithm to one hop count while the other algorithms, especially the ones using rendezvous approaches [19,20], are using multi-hopping (one or more hop count) combined with the use of mobile elements to collect data. Nevertheless we have considered further analysis and comparisons against two recent algorithms [21,22]. In these two recent algorithms the authors proved that their proposed algorithms outperformed other existing ones. In order to compare our algorithm with these two, we adapted their used parameters shown in the Table 6 in our simulation. From Figure 5, it is shown how FNRCA outperformed WRP [21] and Charalampos [22] in terms of network lifetime as the last node in FRNCA died after 1200 s compared to1000 in WRP and 1050 in Charalampos. In WRP 50% of the nodes died after 4500 s, in Charalampos after 4800 s while in FRNCA 50% of its nodes died after 6000 s. This can be mostly contributed to the use of multi-hop communication in WRP and Charalampos which will consume more energy in general and will results in a fast depletion of energy on cluster heads closer to the ferry path. However, in our algorithm checkpoints are just locations where the ferry will stop to collect data from cluster heads that belong to its virtual grid where each cluster head is just one hop count from the checkpoint position they are associated with. Charalampos achieved slightly better results than WRP since it selects cluster heads with higher energy as rendezvous points when using nodes with different initial energy values. However as can be seen from Figure 6, similar results are achieved by both WRP and Charalampos when using the same energy value to start with. In both cases, FNRCA outperformed the two algorithms when using same or different initial energy values. In addition, comparing the graph for FNRCA in Figure 5 and Figure 6 shows minimal changes in its performance regardless of whether the same initial energy value was used by all nodes or uniformly distributed ones were used. This is mainly due to the fact that FNRCA incorporates current energy values in selecting and rotating cluster heads and minimizes multi-hop communication.

**Table 6:** Parameters used in the simulation to compare FNRCA to [21,22].

Notation
N = 200
Initital node energy, Eo = uniformly selected for the nodes from 50–100 J / node
Area = 200 × 200
# Checkpoints = 25
Packet size = 30 Bytes
Data Rate = 40 Kbps
Sensing Radius : r = 20 m
Ferry_speed = 1 m/s

## 5. Conclusions and Future Work

In this paper we have proposed a new efficient data collection algorithm using ferry nodes to collect data from WSN nodes based on a ferry’s path selection. Using this algorithm we achieved efficiency measured by two goals which are minimizing the overall total roundtrip travel time of the ferry and minimizing the overall energy consumed in the whole network. Through simulation we proved the efficiency of our algorithm compared to using traditional multi-hopping method to collect data and using fixed predetermined paths. We showed that nonlinear trajectory achieves a better optimization in term of network lifetime, overall energy consumed and the roundtrip travel time of the ferry. The results of the simulation also showed that using a larger number of checkpoints increases the network lifetime, however, it increases the roundtrip travel time of the ferry.

In our future work, we are considering using multiple ferries to collect data which can be also an improvement over the current proposed algorithm and can decrease the time delay in case of emergency or non-delay tolerant applications. We are also considering changing the algorithm to have a speed controlled fly over ferry instead of stopping at each checkpoints, where the ferry can decrease its speed while flying over checkpoints to collect the data.
